# Nonablative, Noncoagulative Multipolar Radiofrequency and Pulsed Electromagnetic Field Treatment Improves Vaginal Laxity and Sexual Function

**DOI:** 10.1089/whr.2021.0020

**Published:** 2021-07-27

**Authors:** Yegor Kolodchenko

**Affiliations:** Association of Laser Medicine and Cosmetology, Cogerent Laser Clinic, Kyiv, Ukraine.

**Keywords:** female sexual dysfunction, multi-polar radio frequency, pulsed electromagnetic field, vaginal laxity

## Abstract

***Objective:*** This study investigated nonablative/noncoagulative multipolar radiofrequency and pulsed electromagnetic field (RF/PEMF) treatment for vaginal laxity (VL) and its impact on sexual function in parous women.

***Methods:*** This prospective, open-label single-center study enrolled 34 female subjects, 23–59 years of age, with ≥1 vaginal delivery and self-reported VL. Three monthly intravaginal treatments with RF/PEMF energy were performed. Treatment and follow-up assessments included the vaginal health index (VHI), vaginal pH, female sexual function index (FSFI), and VL/sexual satisfaction (SS) and subject satisfaction scores. Mean score and percent improvement over baseline were reported. Subject discomfort/pain was assessed after each treatment.

***Results:*** Total and each individual domain scores of the VHI improved significantly, while vaginal pH levels decreased from baseline to both 1 and 4 months (*p* < 0.01) after the last treatment. FSFI (<0.001), VL (<0.001), and SS (<0.001), including overall satisfaction scores (<0.01), improved post-treatment, with positive effects further sustained until at least 4 months post-treatment. Pain/discomfort post-treatment was reported as none to mild. No noticeable adverse events (AEs) or unanticipated side effects were reported.

***Conclusions:*** Nonablative/noncoagulative multipolar RF/PEMF is safe and is associated with significant 1- and 4-month post-treatment improvements in symptoms associated with VL and sexual dysfunction, as assessed by the VHI, vaginal pH, FSFI, and VL subject satisfaction score. SS and overall satisfaction scores also improved. The treatment was well tolerated with no or little pain, and no adverse events were reported.

Clinical Trial Registration number: NCT04607798.

## Introduction

Vaginal laxity (VL), or a woman's self-assessment of vaginal looseness or tightness, is an underreported and poorly understood symptom of pelvic floor dysfunction, which can have an impact on relationship happiness and sexual function.^1,2^ Further compounding this health issue is the lack of a uniformly accepted definition of VL, or genitopelvic laxity, since it is commonly considered a patient-reported condition deficient in standardization for diagnosis and severity grading that is based on expert consensus or scientific data.^3^ Pelvic floor and vaginal trauma after pregnancy and vaginal childbirth, including stretching of the vaginal introitus, may cause permanent changes, resulting in loss in sexual sensation during intercourse and diminished sexual quality of life.^4,5^ Potential medical consequences associated with vaginal childbirth, including VL, extend beyond the postpartum period and may include stress urinary incontinence, bowel incontinence, pelvic organ prolapse, dyspareunia, and chronic pelvic pain .^4,6,7^ Although underreported, diminished vaginal elasticity and laxity affect 25%–45% of premenopausal women; they have been cited as the most dominant physical changes felt by patients and discussed with physicians following vaginal delivery, with parity being considered a significant risk factor.^2,8,9^ Furthermore, VL may worsen with multiparity, application of forceps, delivery of a large fetus, weight fluctuations, and connective tissue changing due to aging.^10,11^

Treatments for VL include both surgical and noninvasive options. Surgical procedures, including vaginoplasty to tighten the vaginal introitus, have shown improvement in laxity symptoms; however, they are not without risk to the patient.^11,12^ Nonprescription, topical vaginal tightening products lack similar regulatory control compared with prescription products and may impose more harm than good, in addition to requiring continual dosing.^13^ Common, minimally invasive, energy-based treatment options, including the CO_2_ or Er:YAG laser and radiofrequency (RF) devices, are newer technologies and offer an alternative for women who may not wish to undergo invasive surgical procedures due to the downtime and risk involved.^14^ Preliminary data offered by energy-based devices are promising with respect to treatment efficacy of genitopelvic or VL; however, a large disparity does exist between treatment protocols and procedures, making the results difficult for interpretation and determination of consistent effects.^3^

While a range of treatment options exist for VL, their effects on, and in association with, female sexual dysfunction (FSD) are only now starting to be more embodied in the medical literature.^2,15,16^ While there are no specific recommendations regarding the role of RF-based vaginal devices in treating VL, short-term improvements in VL and sexual function have been shown with laser and RF treatments, in which trends in temporary tissue architectural remodeling and integrity, combined with changes in sexual behavior, have been demonstrated.^2,3,11,16^ These objective and subjective parameters established through laxity intervention have been shown to also exhibit short-term improvements related to sexual function.

This study investigated the use of a nonablative/noncoagulative multipolar RF and pulsed electromagnetic field (PEMF)-based device (Venus Fiore™, Venus Concept, Weston, FL) to evaluate its efficacy and safety in treatment of VL and the potential effects on improving sexual function.

## Materials and Methods

### Study design

A single-center, single-arm open-label study was conducted to evaluate the efficacy and safety of a multipolar RF/PEMF system for treatment of symptoms related to VL. IRB approval was received, and the study was carried out in accordance with The Code of Ethics of the Declaration of Helsinki. The trial was registered at clinicaltrials.gov.

### Study population

Healthy Caucasian females, aged ≥19 years, presenting with self-reported VL were invited to participate in study screening. Once informed consent was obtained, it was determined that subjects had a baseline female sexual function index (FSFI) score of ≤26.55, had at least one full-term pregnancy (>36 weeks' gestation, delivered vaginally at least 12 months before enrollment), and engaged in vaginal intercourse at least twice per month. The sample size was based on a previous study that showed a statistically significant change following RF treatment.^11^

### Treatment procedures

Women meeting the inclusion/exclusion criteria received three vaginal treatments using the RF/PEMF device at 1-month intervals and had follow-up (FU) visits at 1 and 4 months after the last treatment. Each treatment was 15 minutes long. The FU visit time frame was chosen so that at 1 month post-treatment, the more immediate treatment effects could be established, while safety and duration efficacy could also be evaluated at a relatively longer term (4 months) after final treatment. Following treatment, subjects were instructed to keep the treated area clean and to refrain from mechanical or thermal injury-causing activities affecting the treatment area.

### Device settings

The internal applicator with disposable cover contains integrated temperature sensors and three pairs of bipolar electrodes (proximal, mid, and distal) responsible for the delivery of multipolar RF/PEMF. The disposable cover stops functioning after 15 minutes to prevent treatment temperature overexposure. The automatic temperature control of the device maintains the preselected, desired, therapeutic temperature range during treatments. Ultrasound gel is used as an RF coupling medium and lubricant for the applicator. During treatment, the vaginal applicator with disposable cover is fully inserted into the vaginal canal where a 15-mm gap housing no electrodes (at the 12 o'clock position) is positioned directly below the urethra, remaining stationary throughout the treatment. The initial treatment energy level was programmed at 50%–70% output for all 3 pairs of electrodes and could be adjusted during treatment based on patient comfort and adverse event (AE) occurrence. Target temperature settings were set to achieve 42°C at the proximal thermometer (closest to the applicator base) and 45°C for the mid and distal thermometers (farthest from the applicator base).

### Outcome measures

Vaginal health evaluation was conducted at baseline and at each study visit by an investigator who was trained to ensure accurate assessment using the vaginal health index (VHI); the VHI includes five vaginal parameters (vaginal fluid volume, moisture, vaginal epithelial integrity, elasticity, and vaginal pH), which sum to a total score of 5–25. A higher score is representative of better vaginal health.^17^ At the baseline visit (and before each treatment as well as at each subsequent study FU visit), subjects were asked to complete the FSFI questionnaire. The FSFI is a validated 19-question survey categorized by six domains of female sexual function: desire, subjective arousal, lubrication, orgasm, satisfaction, and pain.^18^ If the FSFI total score was >26.55 at baseline, the subject could not continue in this study. At each subsequent treatment and FU visit, subject satisfaction was assessed using a 5-point Likert Subject Satisfaction Scale [1 = very unsatisfied; 2 = unsatisfied; 3 = neutral; 4 = satisfied; and 5 = very satisfied]. VL and sexual satisfaction (SS) were self-evaluated by the subjects at each visit after the first treatment using the 7-point Global Response Assessment Scale (GRAS) [from −3 = markedly worse to 3 = markedly improved].^19^

Immediately after each treatment, subject discomfort was assessed using a 10-cm Visual Analog Scale (VAS)^20^ from 0 cm (no pain) to 10 cm (pain as bad as it can be). The VAS score ranges are typically broken down into the following categories: [no pain/discomfort (0–4 mm); mild pain discomfort (5–44 mm); moderate pain/discomfort (45–74 mm); and severe pain/discomfort (75–100 mm)]. Subjects were not permitted to view their previous VAS treatment scores.

The primary outcomes of the study were changes in VHI and FSFI scores. Both these tools are validated global assessment measures in FSD studies. Improvement in VL and sexual function was also assessed based on changes in the VL and SS questionnaire scores, respectively. Overall subject satisfaction with the study was assessed at the end of the trial.

Safety was assessed by documentation of adverse events monitored on an ongoing basis and at each visit following the initial treatment, including the subject's experience of pain or discomfort during and after the procedure.

### Statistical analyses

The percentages, means, standard deviations (SDs), and standard errors (SEs) were calculated for descriptive statistical analyses. Student's paired *t*-test was used to test differences in means, and two-proportion *z*-tests were used to test for statistical significance in the proportion of subjects reporting improvement. A Pearson product–moment correlation coefficient was used to measure the linear association between objective and subjective variables in relation to improvement. Two-sided 95% confidence levels were used where *p*-values <0.05 were considered statistically significant differences. Microsoft Excel, version 2016, was used for data management and statistical analysis.

## Results

Of the 40 women presenting for treatment of VL and screened for the study, 34 were found eligible and willing to participate. The mean (±SD) age and weight of the women who participated in the study was 38.2 ± 7.6 years (age range: 23–59 years) and 59.9 ± 10.6 kg, respectively (Table 1). All subjects completed study procedures and FU visits with no withdrawals and were included in the safety and efficacy analyses.

**Table 1.** Baseline Subject Characteristics and Delivery History (*n* = 34)

Baseline subject characteristics	
Demographic data	
Age (years), mean (SD)	38.2 (7.6)

Age categories	*n* (%)
18–29	4 (11.8)
30–39	20 (58.8)
40–49	6 (17.6)
50–59	4 (11.8)

Clinical data	
Weight (kg), mean (SD)	59.9 (10.6)

Weight categories	*n* (%)
<50	2 (5.9)
50–59	18 (52.9)
60–69	12 (35.3)
≥70	2 (5.9)
Vaginal deliveries, mean (SD)	1.79 (0.64)

Vaginal deliveries, categories	*n* (%)
1	11 (32.4)
2	19 (55.9)
3	4 (11.8)

SD, standard deviation.

**FIG. 1.**
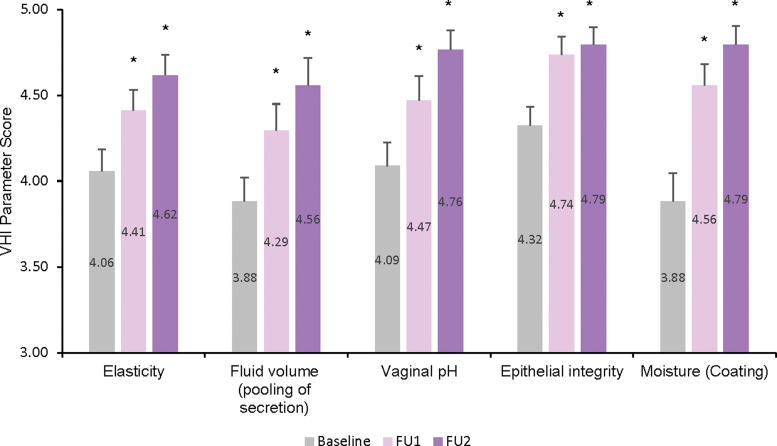
Mean VHI per parameter score: baseline, 1 month (FU1), and 4 months (FU2) after the last treatment (*n* = 34). FU, follow-up; VHI, vaginal health index.

The mean (±SE) baseline VHI score was 20.24 ± 0.58. Statistically significant improvement to 22.47 ± 0.56 was observed at FU 1 (1M FU; *p* < 0.007) and further improvement was recorded at FU 2 (4M FU; *p* < 0.001) with a mean (±SE) of 23.53 ± 0.55. VHI also showed statistically significant increases in the average score of each individual parameter from baseline to 1M FU, with continued improvement at the 4M FU (Fig. 1 and Table 2).

**Table 2.** Mean Vaginal Health Index and Female Sexual Function Index—Domain and Full Scores: Baseline, 1 Month (1M Follow-Up), and 4 Months (4M Follow-Up) After the Last Treatment (*n* = 34)

VHI parameter	Baseline		1 Month post-treatment (1M FU)					4 Months post-treatment (4M FU)				
	Mean	SE	Mean	SE	*p*	% Improvement	% Subjects who improved^a^	Mean	SE	*p*	% Improvement	% Subjects who improved^a^
Elasticity	4.06	0.13	4.41	0.12	<0.001	9	38	4.62	0.12	<0.001	14	56
Fluid volume	3.88	0.14	4.29	0.16	<0.001	11	41	4.56	0.16	<0.001	17	65
Vaginal pH	4.09	0.14	4.47	0.14	<0.001	9	38	4.76	0.11	<0.001	17	65
Epithelial integrity	4.32	0.11	4.74	0.11	<0.001	10	41	4.79	0.10	<0.001	11	47
Moisture	3.88	0.16	4.56	0.12	<0.001	17	62	4.79	0.11	<0.001	23	74
VHI—full score	20.24	0.58	22.47	0.56	<0.007	11	74	23.53	0.55	<0.001	16	88

FSFI domain	Mean	SE	Mean	SE	*p*	% Improvement	% Subjects who improved^a^	Mean	SE	*p*	% Improvement	% Subjects who improved^a^
Desire	3.0	0.1	3.9	0.1	<0.001	28	85	4.1	0.1	<0.001	36	94
Arousal	3.2	0.2	4.1	0.1	<0.001	27	79	4.4	0.1	<0.001	37	94
Lubrication	3.0	0.2	4.2	0.2	<0.001	39	74	4.0	0.2	<0.001	32	65
Orgasm	3.5	0.2	4.2	0.2	<0.001	22	88	4.0	0.1	<0.001	17	79
Satisfaction	3.9	0.2	4.9	0.1	<0.001	25	79	4.9	0.2	<0.001	24	71
Pain	3.3	0.3	4.9	0.2	<0.001	47	85	5.5	0.1	<0.001	65	91
FSFI—full score	19.4	0.90	26.2	0.64	<0.001	35	94	27.0	0.55	<0.001	39	97

^a^ Percentage of subjects who showed improvement over baseline.

FSFI, female sexual function index; FU, follow-up; SE, standard error; VHI, vaginal health index.

An overall nonsignificant reduction in the mean (±SE) vaginal pH level was observed from baseline (4.74 ± 0.10) to 1M FU (4.49 ± 0.09; *p* = 0.08); however, there was significant improvement observed from baseline to 4M FU (4.41 ± 0.07, *p* < 0.05). This reduction indicates an improvement from a basic (higher) pH value to a more acidic (lower) pH value after treatment.

**FIG. 2.**
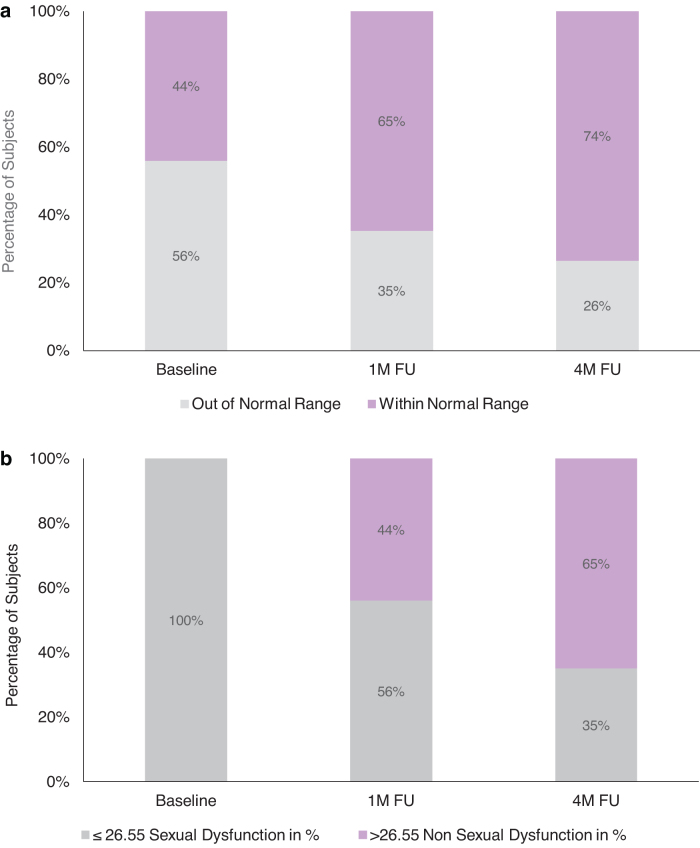
Percentage of subjects pretreatment, at 1 month (1M FU), and at 4 months (4M FU) after the last treatment **(a)** within and outside of the normal vaginal pH range (3.8–4.5) (*n* = 34); **(b)** with sexual dysfunction, as determined by the FSFI score (*n* = 34). FSFI, female sexual function index.

Further improvement was noted in the proportion of subjects who were classified as having pH levels within the normal range (pH values = 3.8–4.5)^21^ after treatment (Fig. 2a). By the 4M FU visit, the percentage of subjects whose pH fell within the normal range increased by almost two-thirds compared with baseline (*p* < 0.05). Additionally, of those subjects with a non-normal range pH post-treatment, 89% (8/9) showed a reduction from baseline pH to a more acidic level even if the normal range was not reached.

Statistically significant improvement from baseline was noted in FSFI scores: at 1M FU, the mean (±SE) scores improved by 35% (baseline: 19.4 ± 0.90; 1M FU: 26.2 ± 0.64; <0.001) and by 39% at the 4M FU (27.0 ± 0.55; <0.001). At baseline, 100% of subjects (*n* = 34) met sexual dysfunction criteria (FSFI score ≤26.55; as required by study inclusion criteria) (Fig. 2b). By the 4M FU, the percentage of subjects classified as sexually dysfunctional dropped to 35% (*n* = 12). Overall, 97% of subjects (*n* = 33/34) experienced improvement from baseline in their combined measure of sexual functioning by the 4M FU. Individual domain scores for desire, arousal, lubrication, orgasm, satisfaction, and pain significantly improved over baseline at both FU visits (*p* < 0.001; Table 2).

A Pearson product–moment correlation coefficient was computed to assess the relationship between the VHI and FSFI. There was a moderate positive correlation between the two variables, *r*(100) = 0.50, *p* ≤ 0.001. Increases in the VHI full score were correlated with increases in the FSFI full score after treatment.

**FIG. 3.**
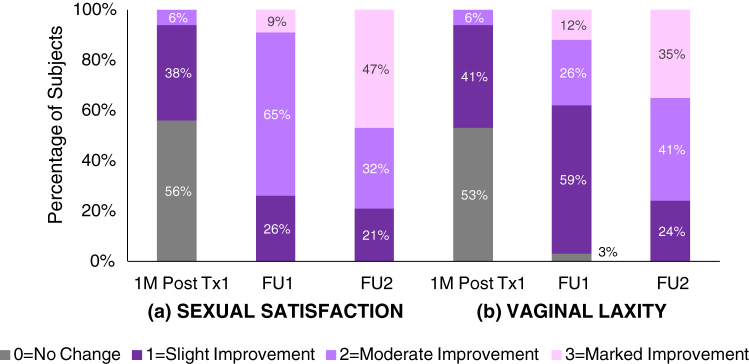
GRAS score distribution for **(a)** SS and **(b)** VL: percent of subjects reporting slight (score of 1), moderate (score of 2), or marked (score of 3) improvement at 1 month after the first treatment and 1 and 4 months after the last treatment (FU1 and FU2, respectively, *n* = 34). GRAS, Global Response Assessment Scale; SS, sexual satisfaction; VL, vaginal laxity.

The subjects' GRAS evaluations for SS and VL showed a significant increase (*p* < 0.001) in reporting of improvement of SS at both the 1M FU and 4M FU visits, compared with 4 weeks after the first treatment. At 1M FU, all subjects had some degree of improvement in their SS: 26% = slight, 65% = moderate, and 9% = marked improvement (Fig. 3). By the 4M FU, subjects expressed further improvement in SS, with most subjects (79%) reporting moderate or marked improvement in SS.

The mean GRAS scores for VL showed almost half of all subjects (47%) indicating some improvement (slight or moderate) after just one treatment, whereas at 1M FU, almost all (97%) expressed improvement: 59% = slight; 26% = moderate; and 12% = marked (Fig. 3). By the 4M FU visit, all subjects reported improvement, with the majority (76%) reporting moderate or marked improvement: 41% and 35%, respectively (significantly different at both 1M FU and 4M FU compared with after the first treatment; *p* < 0.01 and *p* < 0.001, respectively).

**FIG. 4.**
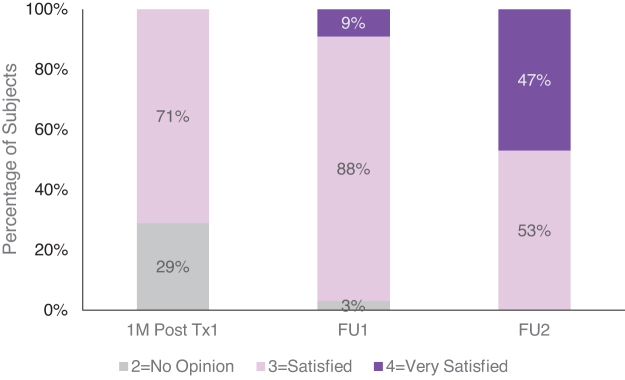
Subject satisfaction score: distribution at 1M post-treatment 1 and 1 month (FU1) and 4 months (FU2) after the last treatment (*n* = 34).

Significant improvement was also seen in subject satisfaction, with mean (±SE) score increasing from 1 month after the first treatment (2.71 ± 0.08) to 1M FU (3.06 ± 0.06; *p* < 0.01) and further to 4M FU (3.47 ± 0.09; *p* < 0.001). After one treatment, 71% reported satisfaction, with the remaining 29% reporting that they had no opinion (Fig. 4). By the 4M FU visit, 100% of the subjects reported satisfaction as a result of their treatments, with almost half of all subjects (47%) reporting being very satisfied. No subjects reported dissatisfaction during the study.

The treatments were well tolerated with no statistically significant changes in reported mean VAS pain scores from treatment to treatment (0.58 ± 0.16 cm, 0.56 ± 0.17 cm, and 0.56 ± 0.18 cm; mean ± SE for treatments 1–3, respectively). Most subjects reported no pain (0–0.4 cm) with all 3 treatments (65%, 71%, and 74%, respectively) with almost all others reporting on the low end of mild pain (0.5–4.4 cm) with the three treatments (32%, 26%, and 24%, respectively).

There were no reports of AEs or unanticipated side effects during the duration of the study.

## Discussion

VL is considered a poorly described and underreported, yet bothersome, condition for women and the actual prevalence is unknown. The progression of VL is considered, in part, to occur naturally in the aging process and research shows that it is also correlated with childbirth.^13^ Moreover, VL has been reported by physicians as the foremost complaint made to them by patients in the postpartum setting.^8,9^ Healthy sexual functioning is an integral part of women's overall general health, and VL in women often generates dissatisfaction with physical sensation and sexual function.^9,22^ The application of energy-based devices for improving VL, and furthermore the subsequent related positive effects on sexual function, is relatively limited in medical literature. Additional research is required so that more conclusive assertions can be made regarding energy-based devices and their positive impact on vaginal tissue integrity and relation to sexual health.

RF+PEMF treatment had positive effects on VL improvement. Objective measures of vaginal health (VHI) saw significant improvement over baseline in mean scores of vaginal fluid volume, improved elasticity, epithelial integrity, and moisture, together with a reduced vaginal pH. The subjective global response scores for vaginal laxity (GRAS-VL) indicated improvement in half of all subjects after just one treatment, with all subjects reporting improvement after all three treatments. Noteworthy is the prolongation of positive outcomes of both VHI improvement and self-reported GRAS-VL responses, with results being maintained and/or further improved to 4 months after final treatment. Millheiser et al. conveyed similar findings using reverse gradient monopolar RF energy through the vaginal mucosa, where 67% of subjects reported improved VL at 1 month, with 87% showing improvement at 6 months.^11^ Their study also confirmed positive outcomes in sexual function scores over the period of 6 months after RF treatment. Histopathologic evidence validating the long-term effects of vaginal RF treatment, helping to explain the mechanism of improvement, was demonstrated by Vanamen et al. (2018). Biopsy evaluations by this group at 120 days post-treatment showed a significant increase in newly formed collagen, demonstrating denser submucosal stroma and increased vascularity, neocollagenesis, neoelastogenesis, and neoangiogenesis.^23^

The vagina normally functions in an acidic environment [pH 3.8 to 4.5].^21^ It is well established in postmenopausal women with vulvo-vaginal atrophy and premenopausal women with estrogen loss (*e.g*., during lactation, cancer treatment, and in smokers) that changes are exhibited in vaginal flora due to increased vaginal pH; a loss in the abundance of protective lactobacilli develops that can increase the potential for bacterial diversity and symptomatic infection.^24,25^ A vaginal epithelium with a rise in beneficial lactobacillus flora, associated with lower pH, is important for maintaining a healthy vaginal state.^21^ While the majority of women enrolled into this study were not of menopausal age, pH results post-treatment proved to be favorable and are worthy of further evaluation. This study saw two-thirds (65%) of all subjects having the pH fall to within the normal range 1 month after three treatments; the positive effect was maintained to 4 months after treatment, with pH of 74% of subjects within the normal range. These results are clinically relevant since the alkalinity of the vaginal flora was reduced to levels typically observed in a healthy vaginal state.^25^ Furthermore, for those subjects whose post-treatment pH did not reduce to normal range values, 89% (8/9) did, however, show a general pH reduction from baseline toward a more acidic level (normal range) after treatment. It is possible that with additional treatments, these subjects' vaginal pH levels may be reduced further.

A stark increase was demonstrated in the proportion of subjects initially considered sexually dysfunctional (combined FSFI score ≤26.55) who improved to a nonsexual dysfunction classification after treatment. Not only were the individual domain scores significantly improved after three treatments, but the maintenance of treatment effects (or further increases—as seen in 5/6 domains) was also seen at 4 months after the last treatment. The global response scores for SS (GRAS-SS), which strengthened the observation of the positive impact of treatment on sexual function, showed at least some degree of improvement in all subjects being reported after three treatments. Treatment effect was maintained (similarly to FSFI outcomes described above) since three-quarters of all subjects reported at least moderate improvement in their SS at 4 months after treatment. It was important to establish a low baseline FSFI value (≤26.55) for study inclusion so that any improvements seen in sexual function could be conveyed as potential enhancements met due to treatment and improvement in VL.

Treatment to the vaginal introitus was well tolerated by subjects and had an excellent safety profile, with no adverse events reported.

Minimally invasive, energy-based treatment options, including the CO_2_ or Er:YAG laser as well as RF devices, offer an alternative to more invasive surgeries for women in treating VL. RF technology is well supported in the literature for application in a wide array of esthetic indications, including cellulite, noninvasive fat removal, and skin-tightening procedures.^26–28^ Recent clinical data suggest that RF may offer a safe and efficient alternative to current treatment modalities for patients seeking to improve the symptoms of VL.^11,14,16,29,30^

The principle of RF treatment for improving VL is based on a sequence of events initiated by heat being introduced into the vaginal wall, which stimulates collagen contraction, neocollagenesis, vascularization, and growth factor production and infiltration; all of these phenomena are believed to play a role in restoring vaginal elasticity and increasing the moisture content of vaginal mucosa.^14,31^ While laser device modalities ablate and coagulate tissue through a wound healing process, monopolar RF devices do not employ ablation; however, they still cause wound healing derived from coagulation.^14^ This mechanism of action was established *via* ovine biopsies^29^ where it was demonstrated that RF treatment increased both fibroblast numbers and activation, and collagen production was found to have occurred in the submucosa at 1 month, persisting to 3 months. The procedure had a favorable safety profile.^29^

Further supporting the histologic evidence, Tadir and colleagues (2018) conducted a comprehensive literature review of clinical data on the efficacy of RF and vaginal health (although related to the genitourinary syndrome of menopause), concluding that available study results on energy-based systems unequivocally demonstrated thickening of glycogen-enriched postmenopausal epithelium, neovascularization, neocollagenesis, increased lactobacilli, reduced pH, vaginal wall tightening, and improved urination control.^31^ The current study reaffirms these findings. Through a distinct mechanism of action, which utilizes multipolar RF in combination with PEMF, a nonablative, noncoagulative stimulating effect provides uniform and controlled delivery of energy distribution. The multielectrode multipolar configuration (vs. monopolar RF and laser devices) avoids physical injury to the tissue used to trigger wound healing and instead utilizes a mechanism of tissue stimulation and not damage.

Although others have demonstrated improvement in the domains of sexual desire, arousal, lubrication, and orgasm in women with baseline sexual dysfunction, including VL using RF,^11,30,32^ the current treatment device is nonablative and noncoagulative and stimulates the tissue in a uniform and controlled delivery of energy distribution. Furthermore, stationary positioning inside the vagina is maintained versus the necessity for interval rotations of the applicator during treatment. Combined, these criteria present more comfort for the patient as well as allow greater ease of use for the treatment provider, while avoiding physical injury in the tissue through a mechanism of stimulation, and not damage. Additionally, there is no downtime experienced post-treatment by the patient, while equivalent efficacy is maintained. This study showed that treatment was well tolerated and normal daily activities could be resumed immediately afterward, in contrast to surgical treatment approaches that run the risk of scar tissue formation, nerve damage leading to fibrosis, dysesthesia, and dyspareunia.^13^

The degree of discomfort/pain experienced during treatment was very mild to none. Most subjects felt no pain during the treatments and reported similar pain (mild or none) after each subsequent treatment, demonstrating that treatment with the device can be administered to patients without significant discomfort. There were no reports of AEs or unanticipated side effects during the study.

There are limitations to this study. The study was performed at a single center, therefore limiting the generalizability of treatment effects on laxity and sexual health after vaginal delivery in the general population. All subjects received the same device treatment, and no negative control or standard of care treatment arm was used. Even though the results of the trial were positive and the treatment was well tolerated and there were no adverse events reported, by conducting a larger controlled (randomized) trial, the positive treatment effects and conclusions could be further strengthened. Despite the study limitations, this study contributes and further supports the medical literature surrounding VL after childbirth and provides further insight related to improvement in sexual health connected with VL and RF/PEMF treatment.

## Conclusions

The results of this study underscore the safety and efficacy of the nonablative/noncoagulative, multipolar RF/PEMF-based therapy for treatment of VL and subsequent positive outcomes on sexual function. The safety profile was exceptionally favorable with no reports of any AEs, significant discomfort, or downtime to the subject associated with treatment.

## Abbreviations Used

AE: adverse event
FSD: female sexual dysfunction
FSFI: female sexual function index
FU: follow-up
GRAS: Global Response Assessment Scale
PEMF: pulsed electromagnetic field
RF: radiofrequency
SD: standard deviation
SE: standard error
SS: sexual satisfaction
VAS: Visual Analog Scale
VHI: vaginal health index
VL: vaginal laxity
